# P-04 - PUBLIC HEALTH MANAGEMENT OF NOTIFIABLE INFECTIONS IN SUDDEN UNEXPECTED DEATHS IN CHILDREN

**DOI:** 10.1093/pubmed/fdag046.077

**Published:** 2026-07-09

**Authors:** Ms Lizzie Murray, Dr Yasmin Ahmed-little, Dr Elizabeth Wareing, Dr Kristina Poole, Dr Caroline Rumble, Dr Elizabeth Stratford, Dr Anna Trelfa, Dr Tanith Palmer

**Affiliations:** UK Health Security Agency; UK Health Security Agency; UK Health Security Agency; UK Health Security Agency; UK Health Security Agency; UK Health Security Agency; UK Health Security Agency; UK Health Security Agency

## Abstract

**Background:**

SUDC (sudden unexpected death in children) cases require careful public health (PH) follow-up. UKHSA Health Protection Teams (HPTs) work in partnership with SUDC paediatricians and local authority PH teams. PH control measures should be implemented swiftly but sensitively whilst coronial investigations are ongoing. The non-specific presentation of general sepsis adds challenges with accurately identifying causative organisms to direct appropriate PH actions. SUDC cases also occur rarely. HPTs may have limited experiences managing deaths, and without careful consideration may default to routine standard operating procedures not always appropriate following a child death. The increase in iGAS (invasive group A streptococcus) infections in children during the national 2022/3 outbreak led to an increase of SUDC cases notified to UKHSA HPTs, highlighting these challenges.

**Objectives:**

To collate learning from recently notified SUDC cases to inform future PH management and ensure case management is streamlined and effective in delivering necessary PH interventions whilst working within the limitations around SUDC cases.

**Methods:**

Recent SUDC cases were reviewed and presented for joint discussion at the annual GM SUDC Study Day. Feedback from this was used to create a joint GM SUDC pathway clearly outlining PH actions required at different stages in case management and the corresponding roles and responsibilities for each team. The pathway was then reviewed at the joint GM SUDC and GM Coroners steering group. Learning from this and subsequent SUDC cases was used to refine the pathway further.

**Results:**

Feedback suggests this pathway has facilitated more effective joint management of SUDC cases, clarifying roles and responsibilities for follow-up PH actions particularly for staff unfamiliar with SUDC case management, reducing the risk of PH actions being delayed or missed.

**Conclusions:**

Joint care pathways across clinical and PH systems can support more effective management for rare and sensitive cases as seen in SUDC scenarios.

